# Ultrafast carbon nanotubes growth on recycled carbon fibers and their evaluation on interfacial shear strength in reinforced composites

**DOI:** 10.1038/s41598-021-84419-y

**Published:** 2021-03-02

**Authors:** A. Salas, C. Medina, J. T. Vial, P. Flores, C. Canales, V. Tuninetti, A. F. Jaramillo, M. F. Meléndrez

**Affiliations:** 1 Department of Mechanical Engineering (DIM), Faculty of Engineering, University of Concepción, Edmundo Larenas 219, 4070409 Concepcion, Chile; 2 Department of Materials Engineering (DIMAT), Interdisciplinary Group of Applied Nanotechnology (GINA), Hybrid Materials Laboratory (HML), Faculty of Engineering, University of Concepcion, 270 Edmundo Larenas, Box 160-C, 4070409 Concepcion, Chile; 3 Departamento de Ingeniería Mecánica, Universidad de la Frontera, Francisco Salazar 01145, 4780000 Temuco, Chile; 4 Unidad de Desarrollo Tecnológico, Av. Cordillera N° 2634 Parque Industrial Coronel, Box 4051, 4191996 Concepción, Chile; 5 UdeC, Edmundo Larenas 219, Concepción, Chile

**Keywords:** Carbon nanotubes and fullerenes, Organic-inorganic nanostructures, Structural properties, Synthesis and processing

## Abstract

The global demand for products manufactured with carbon fibers (CFs) has increased in recent years; however, the waste generated at the end of the product lifetime has also increased. In this research, the impact of the addition of carbon nanotubes (CNTs) on the interlaminated resistance of recycled carbon fibers (RCFs) was studied. In this work, a recycling process of the composite material was applied via thermolysis to obtain the CFs, followed by the growth of CNTs on their surface using the Poptube technique. The recycling temperature were 500 °C and 700 °C; and ferrocene and polypyrrole were used to grow CNTs on CFs surface. CNTs were verified by Raman spectroscopy and scanning electron microscopy (SEM). Finally, to determine the interlaminar resistance, a double cantilever beam (DCB) test was performed. The results indicate that with Poptube technique, CNTs can be grown on RCFs using both impregnations. Thermolysis recycling process at 500 °C allowed CFs without resin residues and without visible damage. The DCB tests showed a decrease in the fracture resistance in mode I loading of 34.9% for the polypyrrole samples and 29.3% for the ferrocene samples compared with the virgin carbon fibers (VCFs) samples with a resistance of 1052.5 J/m^2^.

## Introduction

The recycling of a polymer matrix composite is a complex process because it is composed of two or more materials, e.g., a matrix and fibers, with different characteristics and properties. The processes that exist today are focused on recovering the fiber either chemically or thermally or crushing the entire material, which is known as mechanical recycling^1^. Chemical and thermal recycling processes, which seek to recover matrix fibers using hydrolysis, pyrolysis or a fluidised bed processes^2^, have the potential to reuse the fibers to produce a new part, while mechanical recycling only allows the crushed material to be used as a reinforcement for other parts. During these mechanisms, temperatures vary from 450 to 700 °C in spaces with and without oxygen, where the matrix is decomposed into lighter molecules, turning into gas and releasing the fibers^3^. In this process, the mechanical properties of recycled fibers greatly depend on the temperature at which the process takes place. Geraldin Olilveux et al.^4^, provide a summary of several investigations where changes between − 5 and − 85% of the tensile strength (unique fiber) can be observed when the temperature of the pyrolysis process in the CF varies. The glass fiber, according to the same investigation, undergoes even more with this process, showing poor mechanical properties. Pickering^5^ shows the dependency of the temperature, where it can be seen a decrease of 30% at 600 °C and only a slightly decay at 500 °C not informed quantitatively, as well as a process at 200 °C with a catalyst that had a reduction between 1 and 17% in the tensile strength. Many studies^6,7^ report a fiber carbonization, matrix waste or oxidation, and it is presumed that the decrease of the properties or the lack of adhesion to the new matrix are due to these processes.

All the carbon fibers have a recovering named “sizing”, which protects them during the handling, transportation and manufacture, but also improves the interface or bonding between the fiber and the matrix and also the adhesion to the matrix strongly depends on it. There even exist some special sizings for certain types of resin (matrix). Nevertheless, generally these are only for a “generic” use and not for a specific one^8^. In addition, it is possible that during the recycling process, due to the use of high temperatures, the fiber sizing is lost, as well as the protective coating of the fibers during handling, transport and manufacturing, which at the same time improves the interface between the fibers and the matrix^9^.

Notably, because of the progress in nanotechnology and in particular, the synthesis CNTs, it has been demonstrated that the addition of these nanostructured materials into a matrix strengthens the interface of the composite materials^10–13^ and improves both the interlaminar and fracture resistance^14–17^. However, other studies report problems of agglomeration and difficulties during their addition to the matrix due to their high levels of viscosity, resulting in a decrease in the properties^18–22^. The solution to these issues is found if the CNTs are grown on the fibers. In addition, according to Gorbatikh et al.^23^, a 30% improvement in the interfacial cut resistance (interfacial shear strength, IFSS) is obtained compared to when the CNTs are dispersed in the matrix.

Several methods are used to grow CNTs on surfaces (e.g., fibers) such as chemical vapour deposition (CVD); but, this technique is complex and expensive due to the use of reactors, gases and high temperatures^24^. Another technique that allows these nanostructures to grow uses microwaves, where the growth of CNTs is achieved simply and quickly with ferrocene in the presence of a conductive material in a microwave oven^25–27^. However, this synthesis method works well for VCFs and there is little study on whether it can be used in RCFs. If the surface modification of RCFs takes place with CNTs, then, it is possible to improve the matrix-fiber interface, avoiding abrupt drop in the mechanical properties of the composite materials prepared with them. In the present work, a thermal recycling method was used to recover CFs at 500 and 700 °C. Effect of different types of impregnations on the CNTs growth was studied using poptube method and interfacial shear strength of reinforced composites was evaluated by a double cantilever beam (DCB) test.

## Materials and methods

The material used for this study was TENAX HTA 40/200 tex (3 k) CFs, which was impregnated with a KUKDO YD-114F epoxy resin and KH-813 hardener. The samples were first manufactured by a vacuum-assisted process, with virgin fiber laminates, from which the fibers were then recovered through a recycling process. (5 × 2) cm specimens were cut to undergo the thermal recycling process.

### Fiber recycling process by thermolysis

CFs were successfully recycled by thermolysis under an oxygen atmosphere, for this, a programmable temperature oven THERMOLYNE brand was used. The process was carried out at two temperatures 500 °C and 700 °C for 60 min and a heating ramp of 10 °C/min was used.

### Poptube process for the superficial modification of the RCFs with CNTs

**Figure 1:**
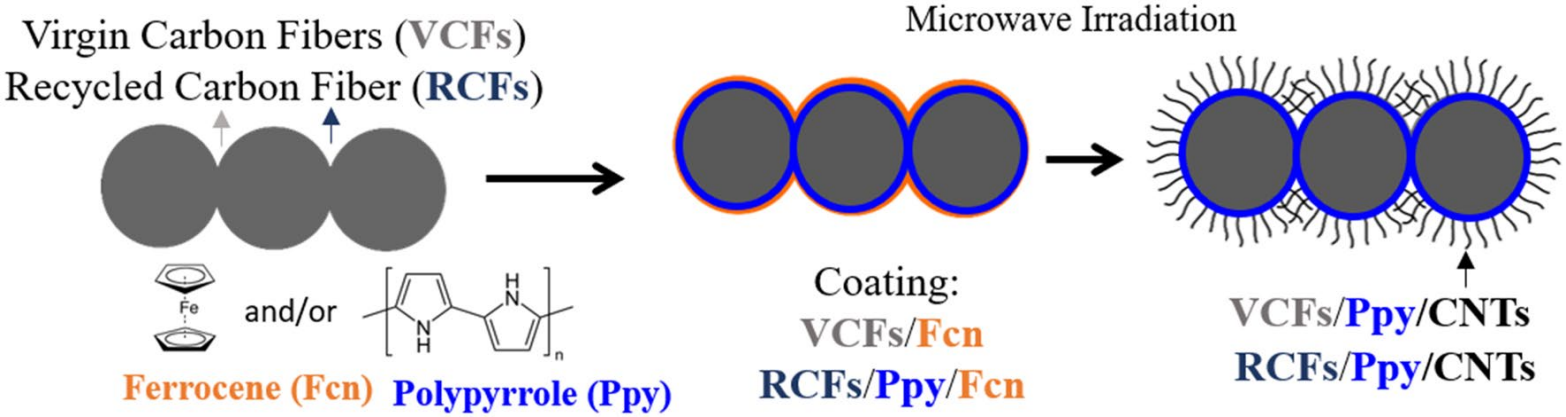
Poptube process scheme, decomposition of a carbon source and growth of microwave-assisted CNTs.

Poptube is a microwave assisted synthesis method to directly heat carbon fibers, providing fast and energy efficient heating. When the fibers are coated with a precursor that is used as a catalyst and carbon source, carbon nanotubes can be grown vertically from the surface of the fiber^28^. This method is novel, efficient and very fast for the growth of CNTs in carbon fibers in large volume. For synthesis, two procedures were carried out for the growth of CNTs: (1) the fibers were impregnated only with ferrocene and (2) the impregnation was carried with polypyrrole and later with ferrocene. All the reagents were purchased from Sigma Aldrich (USA). Pyrrole (98%) and ferrocene (99%) were used for the synthesis and the modified process by Guin et al.^29^ was followed. Procedure 1, consisted of twice impregnating of RCFs with a 0.5 M ferrocene solution in toluene for 15 min. Subsequently, the specimens were vacuum dried at 60 °C for 120 min. Next, the fibers were irradiated by microwaves with a 700 W microwave oven at a frequency of 2.45 GHz for 25 s. In procedure 2, impregnation with pyrrole is carried out with methyl orange^28^. In a typical impregnation, a pyrrole aqueous solution and methyl orange in a 1:10 molar ratio are used, then RCFs are dipped into it for 120 min and after FeCl_3_ aqueous solution in equal volume is added. Mixture is stirred 120 min and left under gentle shaking overnight at room temperature. After the polymerization, RCFs/Polypyrrole are washed with ethanol and dried under vacuum at 60 °C; subsequently, ferrocene impregnation used in procedure 1 was carried out to obtain RCFs/Polypyrrole/ferrocene samples. A schematic of procedure used is shown in Fig. 1.

### Morphological, structural and mechanical characterization

The characterization of the composite material and the CNTs growth from the Poptube microwave-assisted physical method was performed by Raman spectroscopy. The spectra were acquired using a high-resolution confocal LabRamHR Evolution Horiba Jobin Yvon microscope, with a 633 nm Edge laser line excitation source with a power of 13.3 mW. The laser spot was focused on the sample using an optic Objective Olympus 100× VIS and NUV camera (B/S UV 50/50 + Lens F125 D25). In all the generated spectra, the D and G bands were fitted with Lorentzian curves using Origin software and the values of G and D band positions and ID/IG ratios were determined graphically by the fitted curves. So the intensities of the bands (ID and IG) as well as the ID/IG ratio were determined in this new condition.

The morphological characterization CF composites was performed using a scanning electron microscopy (SEM; Joel JSM 6300 LY) instrument at an accelerating voltage of 20 kV. Finally, to study the interlaminar resistance, the double beam cantilever (DCB) test was performed according to the parameters established in the American Society of Testing and Materials (ASTM) D5528-13 standard using a Zwick/Roell brand Z005 universal testing machine. To manufacture the specimens for the DCB test, whose geometry can be seen in “Mechanical tests”. 14 carbon fiber sheets were used and with a non-adhesive sheet between sheet 7 and 8, provided that delamination begins there. The manufacturing was carried out by the vacuum infusion method with a KUKDO YD-114F epoxy resin and KH-813 hardener. To obtain representative results, as recommended by the standard, five tests were carried out for each sample. During the DCB test, the length of the delamination, the advance of the load point and the load must be determined. The speed with which the test was performed was 1–5 [mm/min] as recommended by the standard. Then, the resistance to interlaminar fracture was determined with Eq. (1):

1 $${G}_{1}=\frac{3P\delta }{2ba}$$

where, P is the load, δ is the displacement of the fixed points, b is the width of the specimen, a is the length of the crack. In order to analyze the impact of the modification made to the recycled carbon fiber, specimens made with virgin carbon fibers and recycled carbon fibers without surface modification were also tested. The results of the RCFs with CNTs were compared with VCFs and RCFs without CNTs. Five tests were conducted for each sample according to the standard. Tables 1 and 2 show a summary of the samples to be investigated.

**Table 1:** Summary of the recycling process samples.

Sample	State	Thermolysis process condition
MTV	Recycled	Process up to the vapour temperature
M500	Recycled	Process up to 500 °C
M700	Recycled	Process up to 700 °C
MV	Virgin	–

**Table 2:** Summary of the DCB test samples.

Sample	State
MC	RCFs with CNTs
MR	RCFs (unmodified)
MV	VCFs

## Results and discussion

### Recycling process

**Figure 2:**
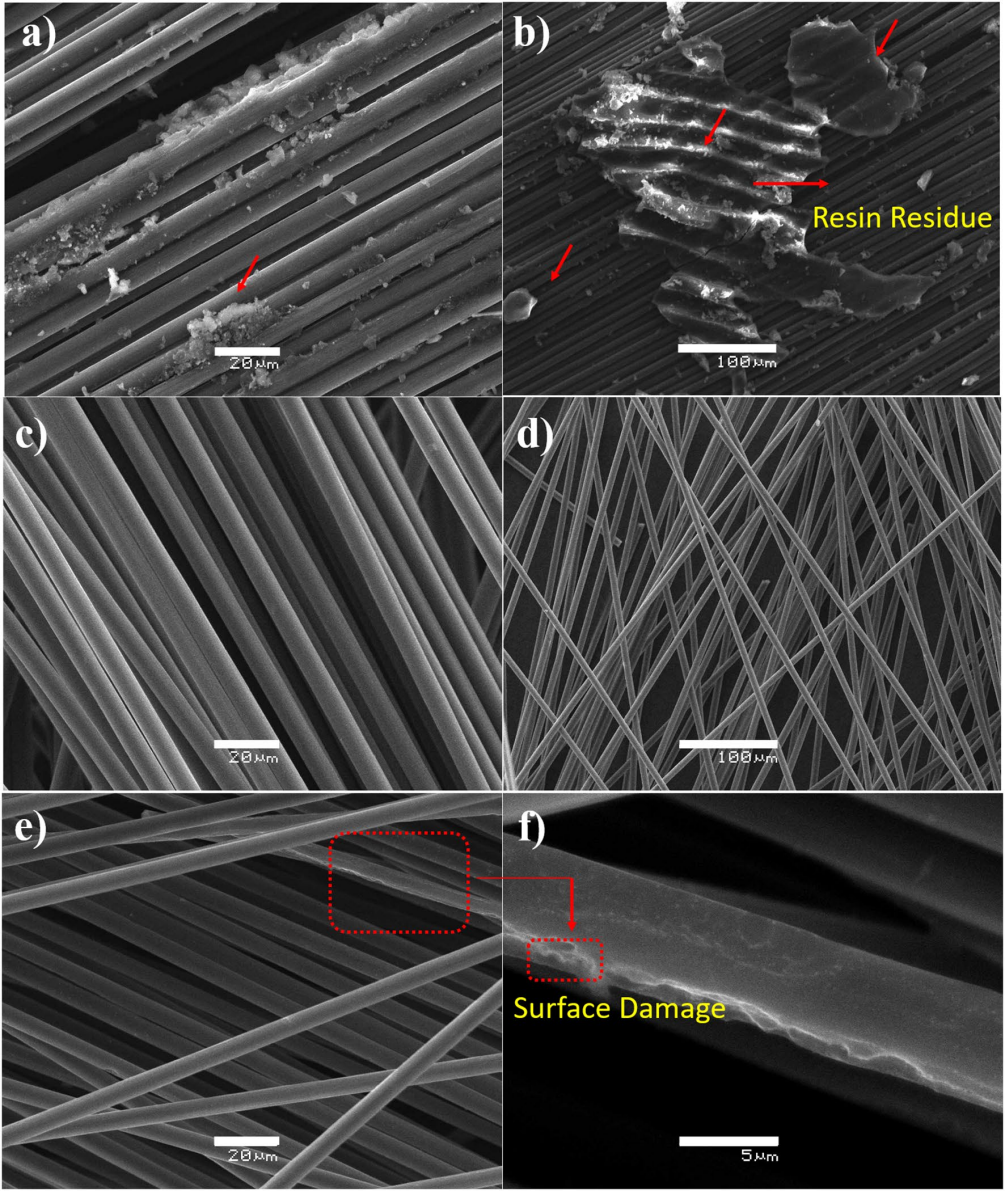
SEM images of CFs recycled by thermolysis (**a**, **b**) until obtaining vapours (onset of matrix decomposition at 350 °C) showing resin residues on its surface, up to 500 °C (**c**, **d**) and 700 °C (**e**, **f**).

In this work, thermolysis method was used for recycling CFs because it has been shown that it does not leave carbonaceous residues on the fiber surface, which can be seen in the SEM micrographs in Fig. 2. In others methods for the composite matrix decompose, such as pyrolysis, that is performed in the absence of oxygen, the fiber surfaces are contaminated with solid char and a subsequent post-pyrolysis treatment (oxidation using air) is required to burn the char to obtain clean fibers and fillers^30^. Post-pyrolysis treatment is mandatory to clean the fiber surface before re-combining the reclaimed fiber with thermoset or thermoplastic composites to synthesis new composite.

Figure 2 shows SEM images of RCFs at different treatment temperatures. Important information on the effect of the thermolysis temperature on the recycled material can be obtained. At the temperature of the vapours (approximately 350 °C), there is still a significant amount of resin between the CF fabric, as shown in Fig. 2a,b. This indicates that the temperature in the thermolysis process is not enough to decompose the entire polymer matrix. These resin residues disappear when the pyrolysis temperature rises to 500 °C as shown in Fig. 2c,d. Finally, when the temperature increases to 700 °C there are no traces of resin, but the fibers was seriously damaged, exhibiting a large number of grooves indicative of partial oxidation on the fiber surface (Fig. 2e,f), similar to that reported by Pimenta et al.^31^. Optimal thermal recycling temperatures typically range from 400 to 550 °C without significantly damaging the fiber. To avoid fiber damage problems, it is recommended that the thermolysis processes used be monitored through controlled temperature with fast heating rates, appropriate residence time and suitable length of raw material^30^.

**Figure 3:**
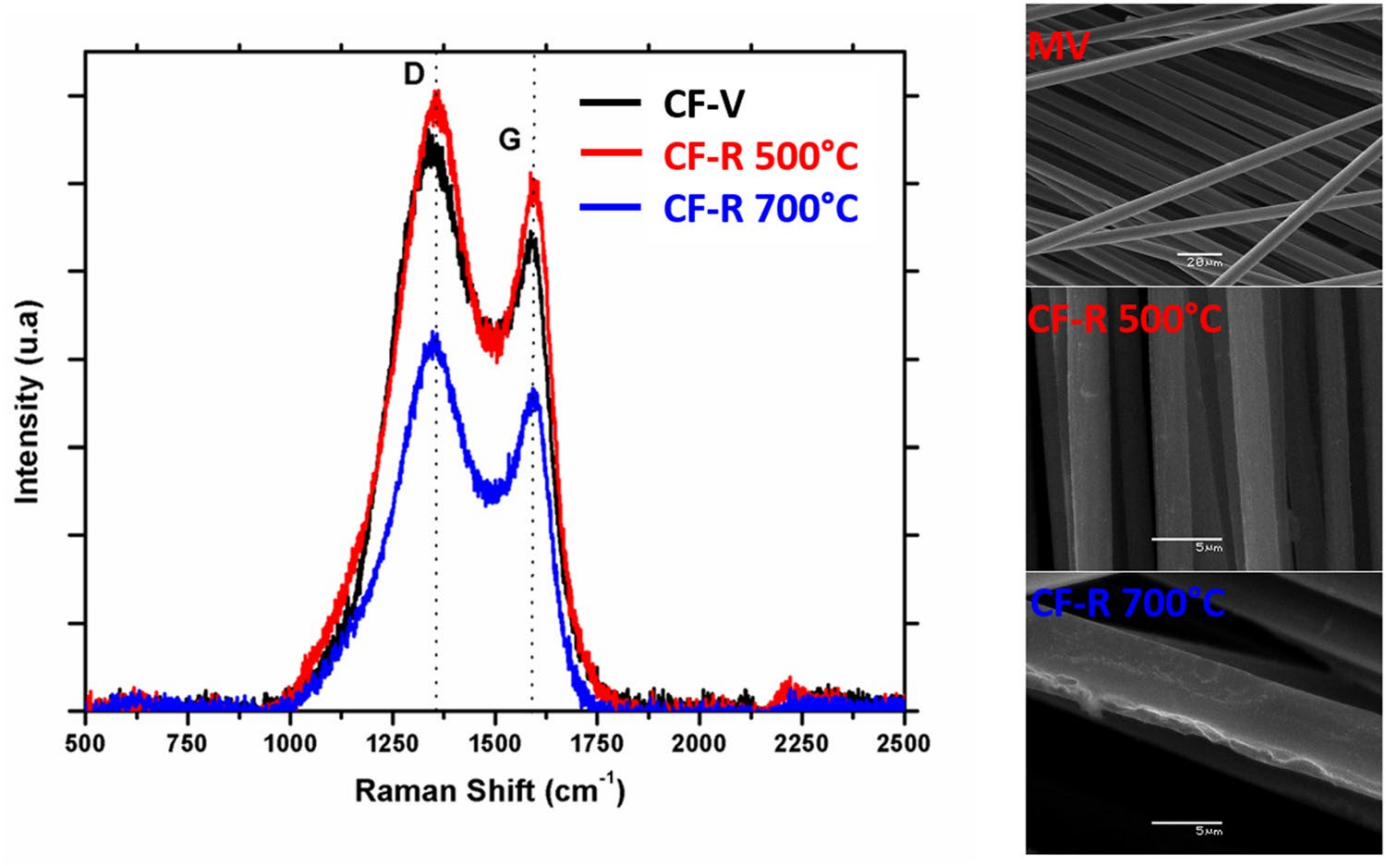
Raman spectroscopy of VCFs and RCFs at 500 °C and 700 °C.

The tensile strength of RCFs has a strong correlation with the intensity ratio of the D and G bands of the Raman spectra (I_D_/I_G_). With an increase in I_D_/I_G_, the tensile strength of RCFs decreases linearly. These observations are supplemented with the results obtained from the Raman spectroscopy. The MT 500, MT 700 and MV samples were analysed by Raman experiments, and the results are shown in Fig. 3. In all samples, the D (~ 1350 cm^−1^) and G (~ 1580 cm^−1^) bands are observed, which is characteristic of graphite or graphene, or both structures^9,29–31^. There are no peaks associated with the presence of resin, but only those related to carbon, which confirms that the fibers are clean or without the evidence of impurities.

Table 3 shows a summary of representative signs of Raman spectra, where both bands shift and intensity ratio (I_D_/I_G_) are found. It is evident that RCFs undergo slight structure modifications during the recycling process and they do not have resin residues on their surface. A smaller integral intensity ratio R (I_D_/I_G_) indicates a higher degree of graphitization, or smaller proportions of the ordered structure of recovered carbon fibers, owing to that the lateral crystallite size of RCFs had become bigger^31–35^. Furthermore, it can be noticed that both G band and D positions band were shifted towards higher wave-number and the D bands became broader after recycling, that can be evidence of microstructural defects of the fibers.

**Table 3:** Raman spectroscopy data of the RCFs.

Sample	D Band shift	G Band shift	I_D_/I_G_	A_D_/A_G_
MV	–	–	1.484	2.73
M500	+ 7.55	+ 11.52	1.376	3.01
M700	+ 5.18	+ 8.48	1.357	3.05

### Poptube process for the superficial modification of the CFs with CNTs

**Figure 4:**
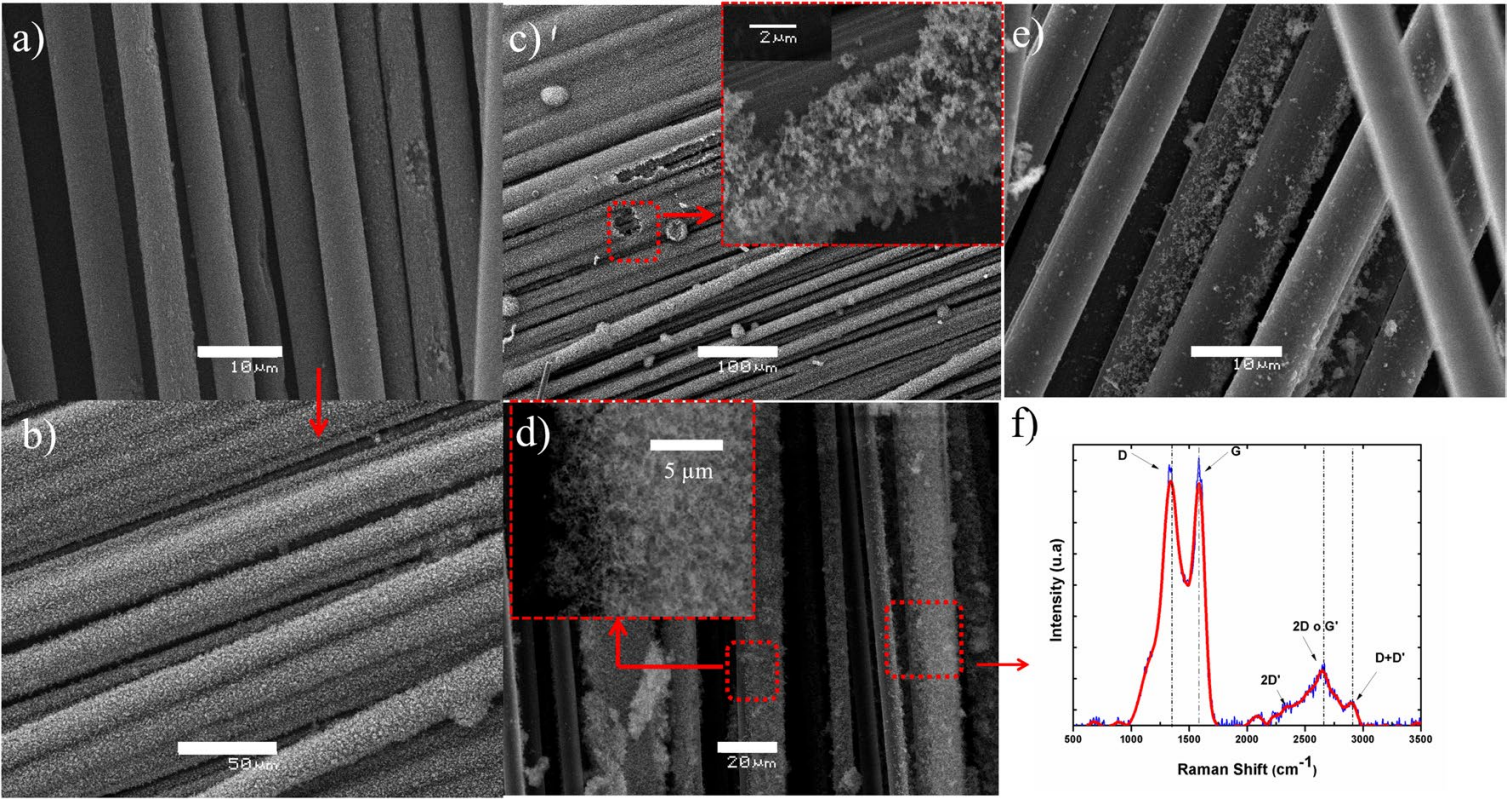
SEM images of CNTs grown via Poptube method on VCFs with polypyrrole/ferrocene (**a**, **b**). CNTs grown on RCFs to 500 °C with polypyrrole/ferrocene (**c**, **d**) and ferrocene (**e**). Raman spectrum of RCFs 500 °C with CNTs (**f**).

Figure 4 shows the SEM images of CNTs growth with the microwave-assisted process, where certain fibers experienced growth of CNTs on their surface. Micrographs in Fig. 4a,b correspond to VCFs, good production of CNTs throughout the CFs is observed, using both polypyrrole/ferrocene and ferrocene impregnation. Micrographs in Fig. 4c–e correspond to CFs recycled at 500 °C with polypyrrole/ferrocene and ferrocene impregnation, respectively. CNTs growth is much less in RCFs without polypyrrole (Fig. 4e), possibly due to loss of conductivity by increased defects, microstructural damage and surface carbonization residues of the fibers. This affects CNTs adherence on RCFs, causing them to detach in some areas (Fig. 4d). Raman spectrum (Fig. 4f) shows bands corresponding to CFs (Band D and Band G), which overlap with those of the CNTs, however, these were verified since the characteristic bands of these nanostructures appear in the spectrum, namely, the addition of the G′ or 2D band (~ 2700 cm^−1^) and D + D′ (2092 cm^−1^)^36,37^.

### Mechanical tests

**Figure 5:**
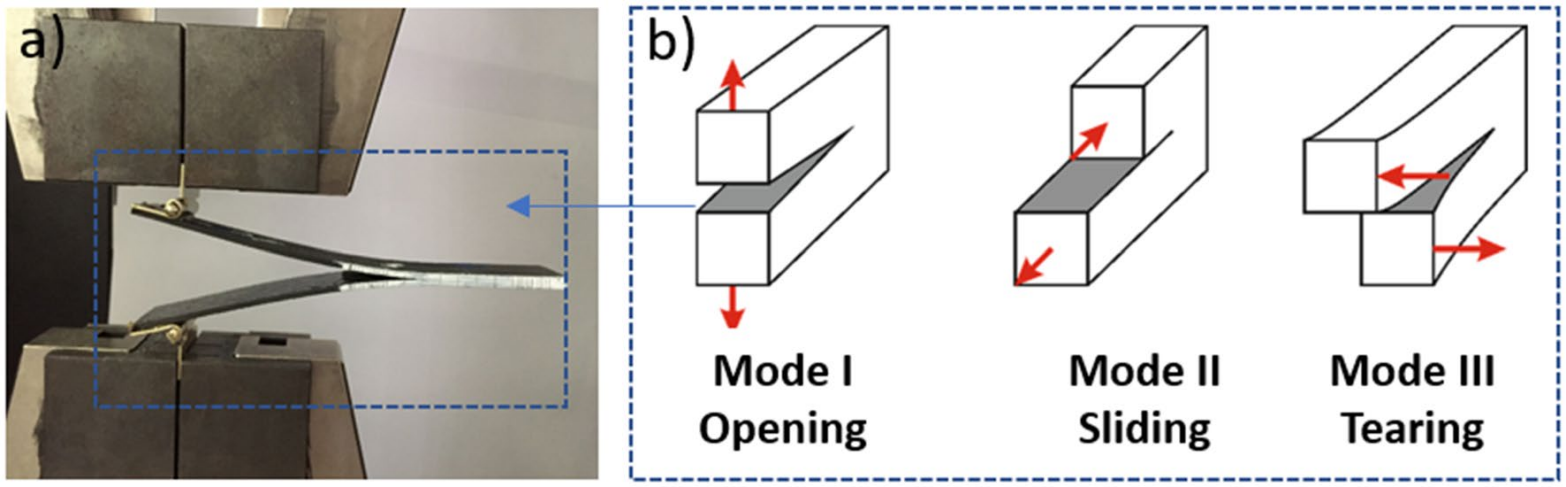
(**a**) Specimen during Mode I test, (**b**) fracture modes and applied loadings.

The damage that usually occurs in laminated composite materials is the separation of two adjacent sheets (plies), this is commonly known in fracture mechanics as interlaminar failure or delamination. There are three fracture modes for interlaminar failures, which depend on the displacement of the crack surfaces: Mode I (opening or tensile mode), Mode II (in-plane shear or sliding mode) and Mode III (out-of-plane shear or tearing mode)^38^. Mode I is used to measure interlaminar fracture toughness in composite materials. A cantilevered double girder is used with a central crack to which a normal load is applied, expanding the crack symmetrically along the girder. It is used as a criterion for damage in the design of composite material structures and between the three modes it provides more information about the matrix-fiber interface. In Fig. 5. Crack propagation caused by different failure modes and loads is illustrated.

**Figure 6:**
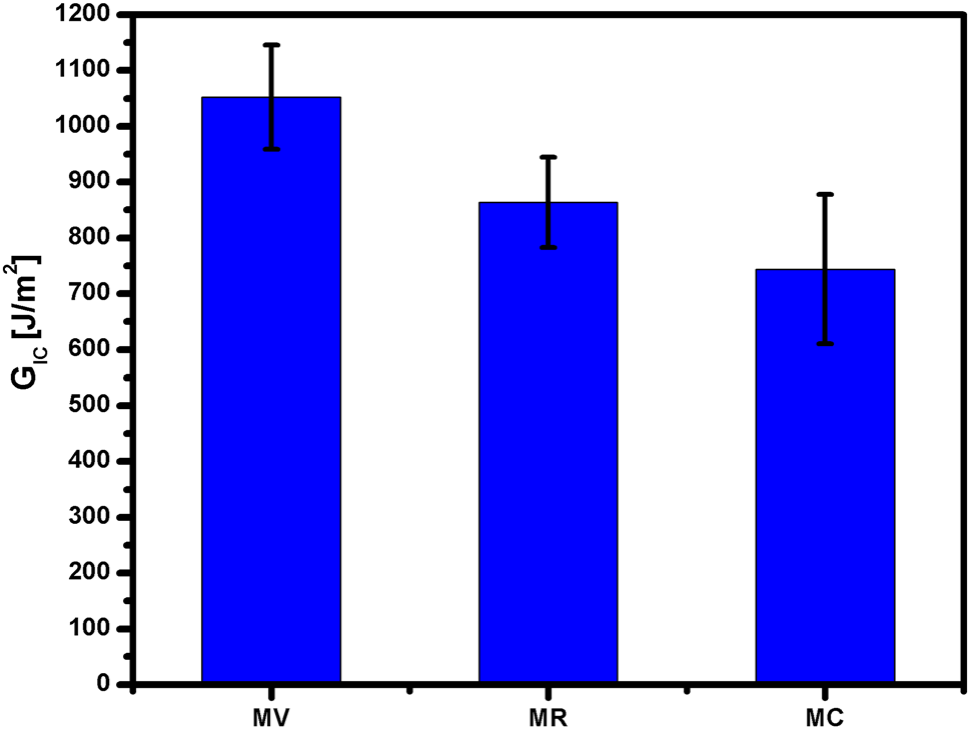
Fracture toughness in mode I loading obtained by the DCB tests.

The results of the fracture toughness in mode I loading obtained by the DCB tests are shown in Fig. 6 and in Table 4. As shown from the results, the recycling process modifies the resistance to fracture in mode I loading, according to that reported in the literature^4,5,39^. This decrease in the mechanical properties is due to a poor interface attributed to the loss of sizing that occurs during fiber recycling, as reported by Dauguet et al.^40^, Yumitori et al.^41^, Paipetis et al.^42^. In the sample with fibers and microwave-assisted growth, a decrease is evident with respect to the RCFs. This result is contrary to that found in the literature regarding the effect of the addition of CNTs to the VCFs on the fracture resistance in mode I loading^43–45^; therefore, the recycling process has a certain negative effect that should be further investigated.

**Table 4:** Summary of the results of the DCB tests.

Sample	G_IC_ (J/m^2^)	Variation (Δ)
MV	1052.48 ± 93.40	–
MR	863.61 ± 80.95	− 17.94%
MC	744.09 ± 133.85	− 29.30%

**Figure 7:**
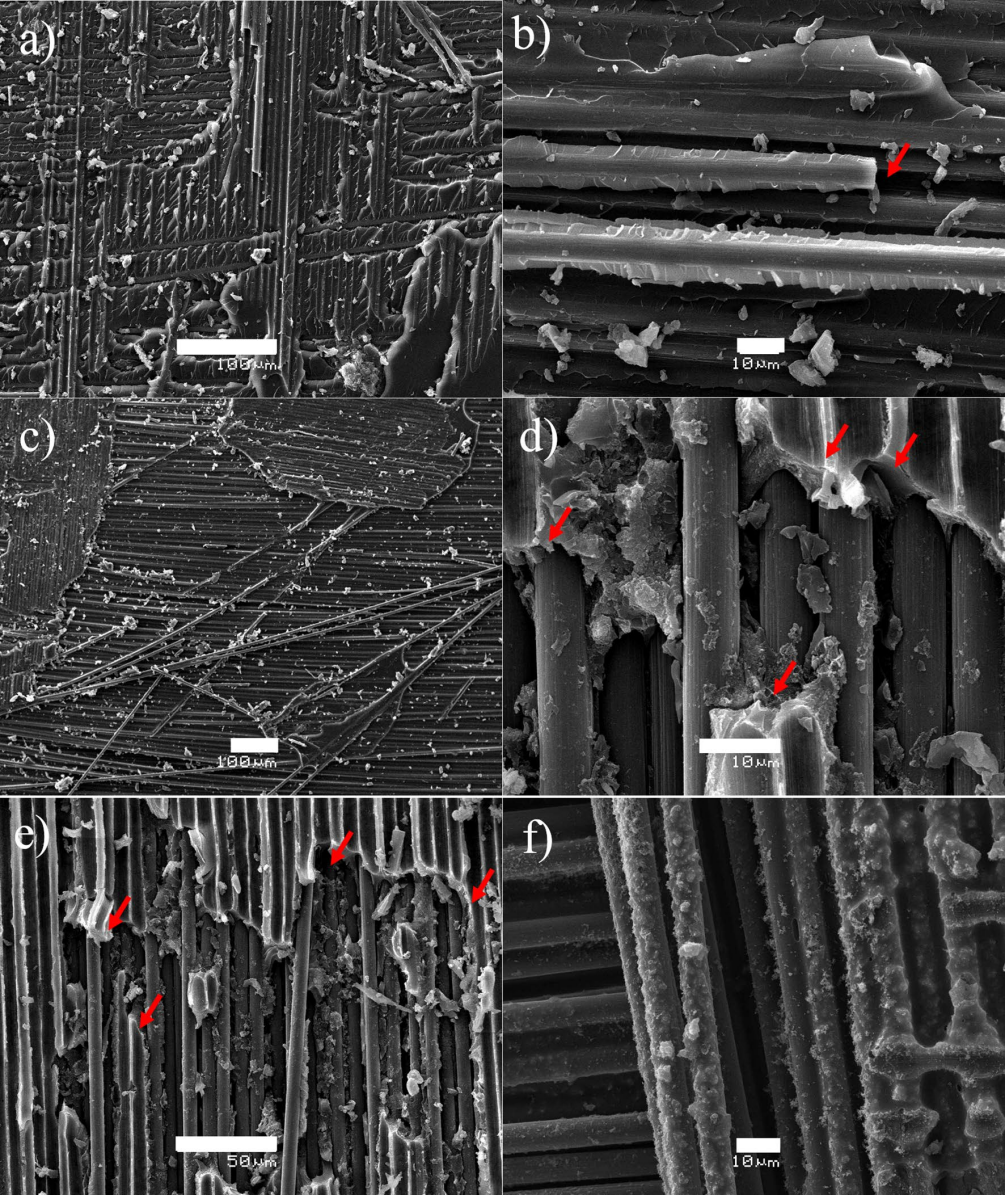
SEM images of the fracture zone of the specimens made with VCFs (**a**, **b**); fibers with good adhesion are observed. The fracture zone of the specimens manufactured with RCFs-500 °C (**c**, **d**); zones with a poor interface zone are observed. The fracture zone of RCFs-500 °C-CNTs (**e**, **f**); CNTs adhered to the fiber and matrix are observed.

**Figure 8:**
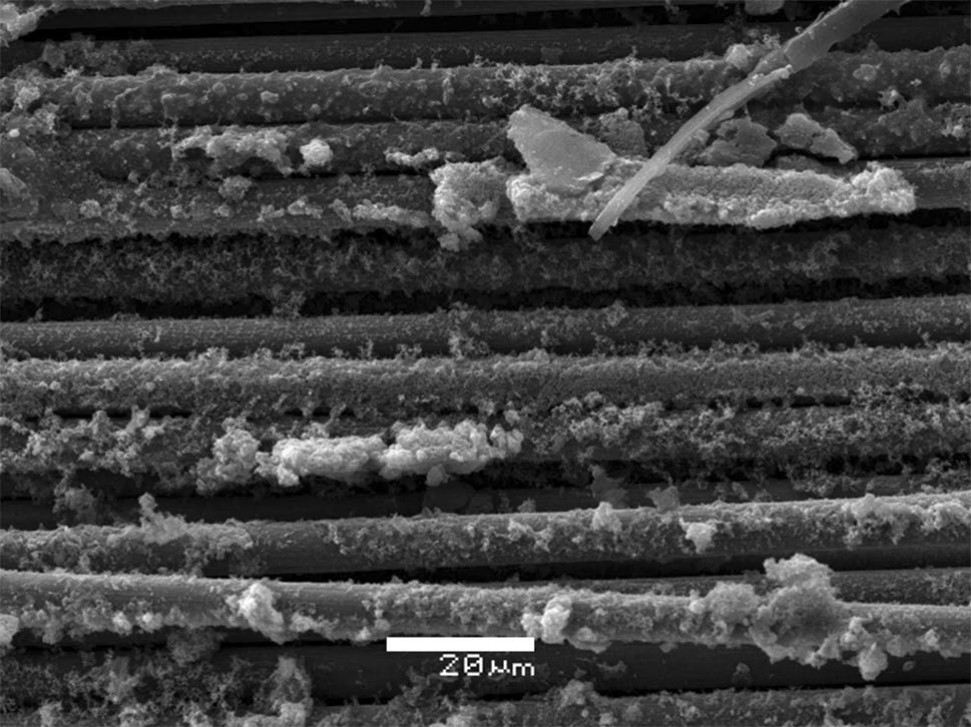
SEM images of the CFR-500 °C-CNTs specimens showing no adhesion to the matrix.

As expected, the images of the fracture zones of the VCFs sample obtained by SEM (Fig. 7a,b) show a type of fragile fracture with a good interface between resin and fibers. The latter can be best observed in Fig. 7b, where the resin is adhered to the fiber. The roughness shown in Fig. 7c is a product of the matrix failure. For the recycled fiber samples shown in Fig. 7c,d, a fracture quite different from the previous one is identified. The roughness disappears, and the fibers have no resin coating, which is the product of a poor resin-fiber interface (Fig. 7c). In Fig. 7d, the marked area shows an approach to the fibers where the complete separation between the matrix and the fiber is more clearly noted. Finally, in the test specimens with CNT growth, different results were obtained. In certain areas of the fractures analysed, no CNTs are observed (Fig. 7e). This result could be because the CNTs did not grow in this particular area or because they separated due to poor adhesion with the fiber at the onset of fracture, similar to that reported by Kim et al.^46^. On the other hand, in certain areas where there was a vast extension of nanoparticles, and two phenomena are observed. The first phenomenon consists of areas of CNTs with a good interface between the fibers and resin (Fig. 7f), where the adherence of CNTs is good with respect to both the fibers and resin^46–49^. However, the second phenomenon appears in areas, such as those shown in Fig. 8, where the CNTs are present on the fibers but without major resin residues, which indicates the good adherence of the CNTs to the fibers but not to the resin.

**Figure 9:**
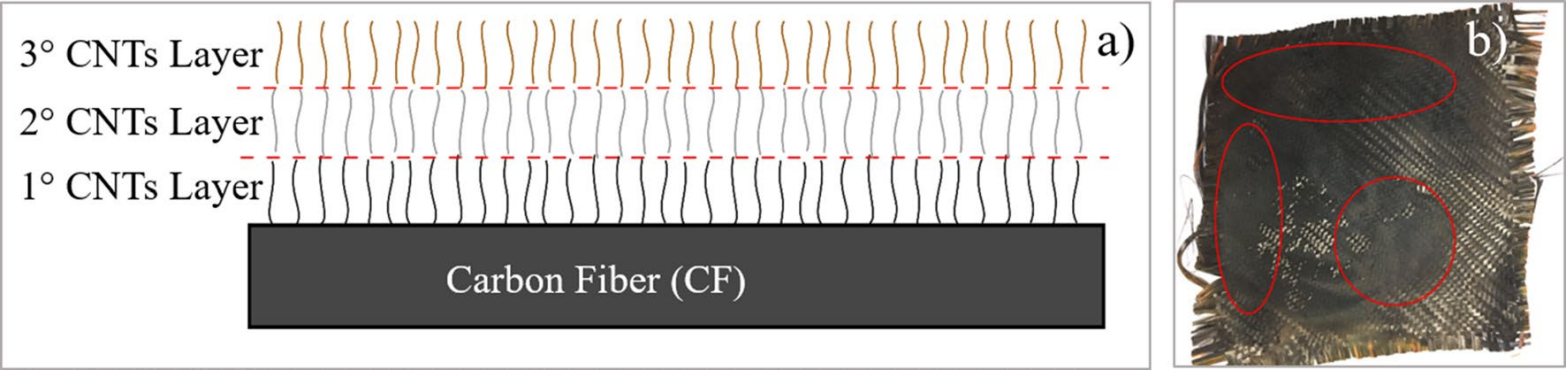
(**a**) Scheme of the possible mechanism for CNT growth by layer on VCFs and RCFs via Poptube. (**b**) CF composite with non-homogeneous growth of CNTs.

The latter may be due to the possible excess of ferrocene over the fibers during the impregnation process, creating a layer that adheres to the fiber, but not in other upper layers, interfering with the interface between the fibers and matrix in the subsequent manufacture of the laminate (Fig. 9a). During the growth process, combustion was generated on the nanoparticles resulting from microwave irradiation. The equipment used has no control over the irradiation power, combustion could not be avoided. As a result of combustion, some areas with a high concentration of nanoparticles remain on the fiber sheets. A scheme for this approach is shown in Fig. 9a. The highlighted areas in Fig. 9b present a higher concentration of nanoparticles. In the areas with the highest concentration of CNTs, the nanoparticles easily detach from the fibers. If the interaction between the CNTs and the fiber is poor or the growth is generated by layers, then the presence of the CNTs reduces the area of contact between the fiber and the matrix. This behaviour hinders the interface, which is reflected by the decrease in the mechanical properties. Similar observations have been reported by other researchers^46,50^.

## Conclusions

The microwave-assisted CNTs growth process allows nanostructures to grow on the surface of RCFs by irradiating the modified fibers with a 700 W microwave oven for 25 s. Alternatively, the process of recycling by thermolysis at 500 °C allows the elimination of the epoxy matrix and rescues the fibers but does not produce major structural changes. For a temperature of 700 °C, surface pitting occurs in certain fiber strands, while at temperatures close to 350 °C (the vapour temperature), the matrix cannot be completely removed. Regarding the mechanical properties, the growth of CNTs on RCFs decreased the resistance to fracture in mode I loading. This decrease was presumably due to the poor adherence of the CNTs to the fiber, the agglomeration of the CNTs layers on the fiber, or both. Further research should focus on improving the recycling process of unmodified CFs and the CFs with CNTs.

## Abbreviations

CFs: Carbon fibers
CNTs: Carbon nanotubes
RCFs: Recycled carbon fibers
DCB: Double cantilever beam
VCFs: Virgin carbon fibers

## References

[1] Giorgini L (2015). Recovery of carbon fibers from cured and uncured carbon fiber reinforced composites wastes and their use as feedstock for a new composite production. Polym. Compos..

[2] Asmatulu E, Twomey J, Overcash M (2014). Recycling of fiber-reinforced composites and direct structural composite recycling concept. J. Compos. Mater..

[3] Ye SY, Bounaceur A, Soudais Y, Barna R (2013). Parameter optimization of the steam thermolysis: A process to recover carbon fibers from polymer-matrix composites. Waste Biomass Valorization.

[4] Oliveux G, Dandy LO, Leeke GA (2015). Current status of recycling of fibre reinforced polymers: Review of technologies, reuse and resulting properties. Prog. Mater. Sci..

[5] Pickering SJ (2006). Recycling technologies for thermoset composite materials-current status. Compos. Part A Appl. Sci. Manuf..

[6] Pimenta S, Pinho ST (2011). Recycling carbon fibre reinforced polymers for structural applications: Technology review and market outlook. Waste Manag..

[7] López FA (2013). Recovery of carbon fibres by the thermolysis and gasification of waste prepreg. J. Anal. Appl. Pyrolysis.

[8] Dai Z, Shi F, Zhang B, Li M, Zhang Z (2011). Effect of sizing on carbon fiber surface properties and fibers/epoxy interfacial adhesion. Appl. Surf. Sci..

[9] Meyer LO, Schulte K, Grove-Nielsen E (2009). CFRP-recycling following a pyrolysis route: Process optimization and potentials. J. Compos. Mater..

[10] Cha J (2017). Improvement of modulus, strength and fracture toughness of CNT/Epoxy nanocomposites through the functionalization of carbon nanotubes. Compos. Part B Eng..

[11] Cha J (2016). Functionalization of carbon nanotubes for fabrication of CNT/epoxy nanocomposites. Mater. Des..

[12] Zheng Y, Chen L (2020). Modification of renewable cardanol onto carbon fiber for the improved interfacial properties of advanced polymer composites. Polymers (Basel)..

[13] Wang C (2017). Enhancing the interfacial strength of carbon fiber reinforced epoxy composites by green grafting of poly(oxypropylene) diamines. Compos. Part A Appl. Sci. Manuf..

[14] Kaddour AS, Hinton MJ (2013). Maturity of 3D failure criteria for fibre-reinforced composites: Comparison between theories and experiments: Part B of WWFE-II. J. Compos. Mater..

[15] Correa E, Gamstedt EK, París F, Mantič V (2007). Effects of the presence of compression in transverse cyclic loading on fibre-matrix debonding in unidirectional composite plies. Compos. Part A Appl. Sci. Manuf..

[16] Canal LP, González C, Segurado J, LLorca J (2012). Intraply fracture of fiber-reinforced composites: Microscopic mechanisms and modeling. Compos. Sci. Technol..

[17] Zheng Y, Wang X, Wu G (2020). Chemical modification of carbon fiber with diethylenetriaminepentaacetic acid/halloysite nanotube as a multifunctional interfacial reinforcement for silicone resin composites. Polym. Adv. Technol..

[18] González C, LLorca J (2007). Mechanical behavior of unidirectional fiber-reinforced polymers under transverse compression: Microscopic mechanisms and modeling. Compos. Sci. Technol..

[19] Juan VS, Fernández E, Pincheira G, Meléndrez M, Flores P (2016). Evaluation of the fill yarns effect on the out-of-plane compressive fatigue behavior for an unidirectional glass fiber reinforced epoxy composite. Compos. Struct..

[20] Romanov VS, Lomov SV, Verpoest I, Gorbatikh L (2014). Can carbon nanotubes grown on fibers fundamentally change stress distribution in a composite?. Compos. Part A Appl. Sci. Manuf..

[21] Godara A (2010). Interfacial shear strength of a glass fiber/epoxy bonding in composites modified with carbon nanotubes. Compos. Sci. Technol..

[22] Pincheira G (2016). Study of the effect of amino-functionalized multiwall carbon nanotubes on dry sliding wear resistance properties of carbon fiber reinforced thermoset polymers. Polym. Bull..

[23] Gorbatikh L, Lomov SV, Verpoest I (2011). Nano-engineered composites: A multiscale approach for adding toughness to fibre reinforced composites. Procedia Eng..

[24] Godara A (2009). Influence of carbon nanotube reinforcement on the processing and the mechanical behaviour of carbon fiber/epoxy composites. Carbon N. Y..

[25] Siegfried M (2014). Impact and residual after impact properties of carbon fiber/epoxy composites modified with carbon nanotubes. Compos. Struct..

[26] Zhang J (2013). Effect of nanoparticles on interfacial properties of carbon fibre-epoxy composites. Compos. Part A Appl. Sci. Manuf..

[27] An Q, Rider AN, Thostenson ET (2013). Hierarchical composite structures prepared by electrophoretic deposition of carbon nanotubes onto glass fibers. ACS Appl. Mater. Interfaces.

[28] Liu Z (2011). Poptube approach for ultrafast carbon nanotube growth. Chem. Commun..

[29] Guin, W. E., Horn, T. & Wang, J. Effects of the poptube approach cnt synthesis process on the tensile properties of carbon fibers and their composites. in *Proc. Am. Soc. Compos. 31st Tech. Conf. ASC 2016* (2016).

[30] Naqvi SR (2018). A critical review on recycling of end-of-life carbon fibre/glass fibre reinforced composites waste using pyrolysis towards a circular economy. Resour. Conserv. Recycl..

[31] Pimenta S, Pinho ST (2012). The effect of recycling on the mechanical response of carbon fibres and their composites. Compos. Struct..

[32] Zhao M (2016). Interfacially reinforced carbon fiber/epoxy composites by grafting melamine onto carbon fibers in supercritical methanol. RSC Adv..

[33] Zhao Q, Wagner HD (2004). Raman spectroscopy of carbon-nanotube-based composites. Philos. Trans. R. Soc. A Math. Phys. Eng. Sci..

[34] Zhan M, Pan G, Wang Y, Kuang T, Zhou F (2017). Ultrafast carbon nanotube growth by microwave irradiation. Diam. Relat. Mater..

[35] Fei J (2018). Grafting methyl acrylic onto carbon fiber via Diels-Alder reaction for excellent mechanical and tribological properties of phenolic composites. Appl. Surf. Sci..

[36] Keszler AM, Nemes L, Ahmad SR, Fang X (2004). Characterisation of carbon nanotube materials by Raman spectroscopy and microscopy—A case study of multiwalled and singlewalled samples. J. Optoelectron. Adv. Mater..

[37] Bokobza L, Zhang J (2012). Raman spectroscopic characterization of multiwall carbon nanotubes and of composites. Express Polym. Lett..

[38] Saadati Y (2020). A study of the interlaminar fracture toughness of unidirectional flax/epoxy composites. J. Compos. Sci..

[39] Kim KW (2017). Recycling and characterization of carbon fibers from carbon fiber reinforced epoxy matrix composites by a novel super-heated-steam method. J. Environ. Manage..

[40] Dauguet M, Mantaux O, Perry N, Zhao YF (2015). Recycling of CFRP for high value applications: Effect of sizing removal and environmental analysis of the Super Critical Fluid Solvolysis. Procedia CIRP.

[41] Yumitori S, Wang D, Jones FR (1994). The role of sizing resins in carbon fibre-reinforced polyethersulfone (PES). Composites.

[42] Paipetis A, Galiotis C (1996). Effect of fibre sizing on the stress transfer efficiency in carbon/epoxy model composites. Compos. Part A Appl. Sci. Manuf..

[43] Wicks SS, de Villoria RG, Wardle BL (2010). Interlaminar and intralaminar reinforcement of composite laminates with aligned carbon nanotubes. Compos. Sci. Technol..

[44] Kepple KL, Sanborn GP, Lacasse PA, Gruenberg KM, Ready WJ (2008). Improved fracture toughness of carbon fiber composite functionalized with multi walled carbon nanotubes. Carbon N. Y..

[45] Zhang H, Liu Y, Kuwata M, Bilotti E, Peijs T (2015). Improved fracture toughness and integrated damage sensing capability by spray coated CNTs on carbon fibre prepreg. Compos. Part A Appl. Sci. Manuf..

[46] Kim H, Oh E, Hahn HT, Lee KH (2015). Enhancement of fracture toughness of hierarchical carbon fiber composites via improved adhesion between carbon nanotubes and carbon fibers. Compos. Part A Appl. Sci. Manuf..

[47] Li F (2018). Effectively enhanced mechanical properties of injection molded short carbon fiber reinforced polyethersulfone composites by phenol-formaldehyde resin sizing. Compos. Part B Eng..

[48] Medina C (2017). Multiscale characterization of nanoengineered fiber-reinforced composites: Effect of carbon nanotubes on the out-of-plane mechanical behavior. *J. Nanomater.*. Compos. Part B Eng..

[49] Medinam C (2016). Comparison of push-in and push-out tests for measuring interfacial shear strength in nano-reinforced composite materials. J. Compos. Mater..

[50] De Riccardis MF (2006). Anchorage of carbon nanotubes grown on carbon fibres. Carbon N. Y..

